# In Defence of Causing Patients to Worry: Ethical Issues in the Communication of Diagnostic Uncertainty

**DOI:** 10.1111/bioe.13436

**Published:** 2025-06-11

**Authors:** Caitríona Cox, Zoë Fritz

**Affiliations:** ^1^ Strangeways Research Laboratory, THIS Institute University of Cambridge Cambridge UK

**Keywords:** communication, diagnostic uncertainty, doctor–patient relationship, ethics, worry

## Abstract

Doctors are often motivated by a desire to avoid causing their patients worry. In this paper, we provide a defence of disclosing diagnostic uncertainty information to patients, even if such disclosures are worrying. We first consider whether making a patient worry harms them, arguing that worry can be harmful in some—but not all—situations. Although worry is an aversive emotion, sometimes, worry can be beneficial (e.g., if the worry drives adaptive behaviours that are ultimately good for the patient's well‐being). In contrast, worry that is excessive, or is related to events outside the patient's control, can be considered harmful. Even if worry *is* harmful, communicating worrying information can still sometimes be justified—for example, by applying a consequentialist harm–benefit analysis to consider whether the other benefits of the disclosure (broadly defined) might outweigh the harm created by the worry. We summarise the growing empirical evidence that suggests that patients often prefer their doctors to communicate transparently throughout the diagnostic process, even if the acknowledgement of serious but uncertain diagnoses induces some worry. We do, however, note the difficulty in predicting how an individual patient will respond to the disclosure of potentially worrying information (as the preference for greater communication of diagnostic uncertainty may not be universal). We conclude that a holistic consideration of the expected consequences of communication—including self‐assessment by the doctor to avoid unwitting bias or unwarranted projection of their own values—often supports the communication of diagnostic uncertainty information, *even* if it worries the patient.

## 1 Introduction

Doctors are often the conveyers of bad news: of diagnoses that may lead to death or require burdensome treatment; of procedures that may be painful and carry risks of serious injury; and of advice that lifestyles need to be changed. It is perhaps not surprising, then, that doctors often seek to avoid causing worry whenever possible. If there is something that they do not *need* to tell a patient, particularly if it might cause worry, they often reason that it is better left unsaid [1, 2, 3, 4].

The ethical permissibility of non‐disclosures in the doctor–patient relationship has been examined in various contexts: regarding prognosis [5, 6, 7], risks of treatment [8] and medical errors [9]. Therapeutic privilege describes the withholding of information if there is a reasonable concern that its disclosure will be seriously detrimental to the patient [8, 10, 11]. In this sense, it represents a limited endorsement of paternalism [8, 10, 11]. It has some recognition in the UK by the General Medical Council (GMC), who state that there are ‘exceptional circumstances in which you may decide not to share all relevant information’ [12]. They highlight that this should only occur in situations where the doctor feels that the disclosure will cause serious harm (not simply that the patient might become upset, or decide to decline treatment).

Over the last few decades, there has been a clear shift in favour of greater information disclosure in the doctor–patient relationship [13]. Fifty years ago, for example, it was common to not tell patients about a terminal cancer diagnosis [14]. In the United Kingdom, until 2014, it was normal to not tell patients that doctors had made a clinical decision to not perform cardiopulmonary resuscitation in the event of their cardiac arrest [15]. Such non‐disclosures are generally now considered impermissible: there are few, if any, situations when we would consider it acceptable for a doctor to withhold information about a definite diagnosis or management plan (however worrisome or distressing that information may be for the patient). There are, however, some situations where the ethical acceptability of withholding potentially distressing information is less clear—for example, when the diagnosis, prognosis or management plan is not yet definite.

One example is the communication of uncertainty in diagnosis, including discussion about alternate diagnoses that are being considered. The diagnostic process is often complex and can be protracted, and it is common for doctors to not be completely certain about the diagnosis. Frequently, there is one diagnosis that is considered the most likely (the ‘working diagnosis’), alongside a list of other potential diagnoses (the ‘differential diagnosis’). Some of these differential diagnoses may be very unlikely, but potentially serious.

Various conceptualisations of diagnostic uncertainty have been suggested. Bhise et al. describe it as the ‘subjective perception of an inability to provide an accurate explanation of the patient's health problem’, [16] while Dahm at al. discuss how a more patient‐centred definition might consider uncertainty in terms of ‘any statement made by a provider that either directly or indirectly indicates uncertainty to a patient’ [17]. Importantly, diagnostic uncertainty communication can be explicit (when a clinician directly informs a patient that they are not sure as to the cause of their symptoms, or that the diagnosis being considered is uncertain) or implicit (when a clinician discusses alternative diagnoses, or when they use ‘hedging’ words to indirectly signify that the diagnosis is not definite) [17].

The degree to which the patient versus the doctor perceives the diagnosis to be uncertain may differ, particularly when the clinician does not explicitly communicate this information. Different approaches to communication may have different effects—for example, explicit versus implicit communication may result in different perceptions of the diagnosis (and the uncertainty therein), and accordingly, different emotional and cognitive patient responses [18]. Doctors—particularly those working in specialities dealing with undifferentiated acute presentations—may be well accustomed to managing uncertainty in diagnosis. Indeed, in acute settings, the doctor's focus is often on ruling out potentially life‐threatening diagnoses (as opposed to reaching a definitive diagnosis). In contrast, patients often expect a definite diagnosis, and may have different (and at times unrealistic) expectations about what is achievable in acute settings [19].

When deciding how to communicate with patients in situations of diagnostic uncertainty, doctors must consider a range of factors. Greater provision of information—including acknowledgement of diagnostic uncertainty and discussion about differential diagnoses—could be helpful in promoting patient agency and facilitating shared understanding. Such potential benefits need to be balanced against the possible negative consequences of the disclosure, including patient distress/anxiety. Doctors may balance these competing considerations differently, and will often reach different conclusions according to the specific clinical situation. There is, however, a tendency to particularly value the communication goal of *minimising patient worry* when it comes to diagnostic uncertainty communication. For example, a recent vignette study demonstrated that doctors do not always explicitly disclose diagnostic uncertainty to their patients, often motivated by a desire to avoid causing worry [20].

We argue that this practice—of not acknowledging diagnostic uncertainty and not disclosing the possibility of a serious alternate diagnosis in the name of avoiding patient worry—should be challenged. Just because information is worrying is not alone reason to withhold it from patients: in many situations, the disclosure of diagnostic uncertainty information can be ethically justified, even if it induces patient worry.

In what follows, we discuss whether worry is harmful, concluding that in some—but not all—situations it can be. We then argue that even if worry is harmful, communicating worrying information can still be justified by an appeal to other ethical arguments in favour of the disclosure. Applying this to the communication of diagnostic uncertainty, we examine the empirical evidence to better understand the impact of (not) communicating it to patients. We conclude that even though diagnostic uncertainty communication can induce worry, the benefits to the disclosure (and the harms of nondisclosure) often still favour communication of the information. We finally address some practical challenges in diagnostic uncertainty communication, in particular, the difficulty in predicting how an individual patient will respond to the disclosure of potentially worrying information.

## 2 Is Worry Harmful?

Doctors' desire to avoid causing worry is embedded in a belief that causing worry constitutes a harm simpliciter; this is the first thing that we challenge.

Although the concept of ‘non‐maleficence’ is widely evoked in the bioethics literature, the question of what precisely constitutes a harm is infrequently addressed. There is, however, extensive philosophical and legal literature examining this question [21, 22, 23]. A range of different approaches have been proposed [24]. Feinberg's counterfactual comparative account [25], although not beyond criticism [23, 26], is widely accepted [27, 28, 29]. This is the account that we will apply here. It states that an event e harms a subject s if, and only if, s would have been better off in the absence of e [24]. It is an account of ‘overall, all‐things‐considered harm’, taking into account the ways in which someone is worse off after an event has occurred [26].

Determining if worry leaves someone in an ‘overall worse‐off state’ requires a consideration of both its negative and positive effects [30, 31, 32]. Worry can be both functional and dysfunctional [30, 33]. It has the potential to act as a driver of adaptive behaviour—this has been explored in a healthcare context, for example, cancer‐related worry as a predictor of engagement with screening programmes [34, 35]. Doctors may deliberately increase a patient's worry about their symptoms to encourage them to follow medical advice; as one GP in an interview study noted, ‘[i]t's never nice to frighten people but I think under certain circumstances you probably have to, to a certain extent’ [36] Ultimately, in some situations, a degree of worry may be appropriate and indeed helpful: as Sweeny asserts, ‘worrying the right amount is better than not worrying at all’ [31]. Worry is not necessarily harmful if it provides an overall benefit by guiding adaptive or protective behaviours—we label this ‘appropriate worry’.

Returning to our area of interest, if a doctor communicates diagnostic uncertainty, even if that information causes worry, it might not harm the patient if the worry itself drives useful healthcare‐seeking behaviours. If a patient is informed about the possibility of an alternate unlikely but serious pathology, they may worry. But this worry (generated by the disclosure of diagnostic uncertainty) might drive re‐presentation in the event of persistent or worsening symptoms [37]. The ‘appropriate worry’ could therefore have an overall positive impact on the patient's well‐being. Worry, then, does not cause harm simpliciter.

There are, however, situations when worry can be considered harmful. It can impair sleep [38] and memory [39]; it has been associated with poor health outcomes (including cardiovascular disease [40, 41]) and reduced quality of life [42]. When worry occurs in response to events or outcomes that are outside the worrier's control, it can be ‘futile and needlessly unpleasant’ [31]. We argue that worry can be harmful if it is excessive (particularly if it is out of proportion to the risk of the future events occurring), if it significantly impairs functioning or if it occurs in the absence of protective behaviours that can be taken to reduce the risk of future events. Worry can thus have an overall negative impact on well‐being: we label this ‘harmful worry’. In the next section, we will discuss in what context causing even this kind of harmful worry may be justified.

## 3 Is the Disclosure of Information That Induces Harmful Worry Still Sometimes Justified?

Doctors should avoid causing patients harm—including causing harmful worry. This does not, however, mean that doctors must avoid it at all costs: the duty to avoid causing harmful worry is not absolute, and in certain situations, it may be overridden by other competing ethical considerations.

The existing literature typically involves an examination of the moral arguments in favour versus the moral arguments against disclosing potentially worrying information [43, 44, 45, 46, 47], taking into account the specific clinical context (including the nature, seriousness and urgency of the information being transmitted/withheld) [11]. Many accounts adopt a broadly consequentialist approach: when it would be morally justifiable to disclose worrying information to a patient turns on a calculation of the overall harms versus benefits of the disclosure [44, 48]. The doctor must consider the consequences of disclosing the information and also the consequences of not disclosing the information. Determining the right course of action involves weighing these up in a ‘harm–benefit calculus’ [43]. Importantly, the harms and benefits can be broadly defined and must take into account not just the consequences for the individual patient but also wider societal implications. As Sokol notes, ‘harm is not limited to medical harm, but includes a range of other harms, from psychological distress to a breach of trust by the medical profession’ [43].

Respect for autonomy is frequently highlighted as an important benefit to disclosing information to patients.^1^ Various conceptualisations of autonomy have been applied in bioethics [49]; Beauchamp and Childress' conceptualisation is perhaps the most widely used (according to which, autonomy is the right to self‐governance, to act in accordance with a self‐chosen plan with intention, understanding and noninterference) [50]. Different accounts vary not only in how they define but also in how they value autonomy: some highlight its instrumental value [51], while others highlight its intrinsic value [52]. Accounts that emphasise its intrinsic value (e.g., many Kantian accounts) hold that autonomy is a cardinal good in and of itself, even if exercising one's autonomy might lead to ‘bad’ outcomes. In contrast, those who emphasise its instrumental value argue that respecting a patient's autonomy will often have good consequences for the them (not least because they are often in the best position to know what would promote good in their own lives) [53, 54, 55]. As Gillon argues, respecting autonomy by avoiding deceit is ‘defensible from several moral perspectives, including those primarily concerned with maximising welfare, provided that welfare is not assessed simplistically on the basis of mere consideration of a patient's immediate distress on being told dire news’ [55]. It is uncontroversial to state that autonomy is a valued concept in bioethics; we argue that whichever conceptualisation of autonomy is being applied, the provision of information to promote patient autonomy can be considered one of a range of potential benefits to information disclosure.

Ultimately, the harm–benefit calculation as applied to information disclosure must involve a broad and holistic definition of both harm and benefit. Some have focused on developing a process to help doctors make such calculations in practice [11, 56]. For example, Sokol developed a ‘deception flowchart’ that prompts the doctor to balance the justifications for and objections to both lying and non‐lying deceptions [56] Richard et al. build on this by proposing a multi‐step reflective process, requiring the doctor to explicitly consider the both the impact on the patient's well‐being and the detrimental consequences of denying a patient's right to know the ‘truth’ [11].

In summary, even if worry is harmful, communicating worrying information can still sometimes be ethically justified—for example, by considering whether the other benefits of the disclosure (broadly defined, including benefits such as the promotion of patient autonomy) outweigh the harm created by the worry. When making judgements about the acceptability of (not) disclosing such information, it is thus necessary to understand the consequences of the disclosure.

## 4 What Are the Consequences of (Not) Communicating Diagnostic Uncertainty?

We have so far argued that although doctors should in general avoid disclosing information that creates harmful worry, such disclosures may sometimes still be justified through a consideration of the benefits to the disclosure (and the harms of nondisclosure). Applying this to diagnostic uncertainty communication, we must consider the effects of its (non) communication to patients.

### 4.1 The Effects of Communicating Diagnostic Uncertainty to Patients

There is reason to believe that honest disclosure of information within the doctor–patient relationship—even of distressing or worrying information—can be beneficial. In palliative care, research has explored the impact of disclosing prognostic information to patients with advanced, life‐limiting illnesses [5, 6, 57]. Although historically doctors expressed concerns that such discussions would have negative effects on patients, studies show that realistic and honest disclosure is preferred by patients and is seen as important in facilitating informed decision‐making and building trust [5, 58, 59]. Indeed, perceived physician honesty has been demonstrated as important in promoting trust within the doctor–patient relationship [60].

Less research has specifically examined the impact of disclosing diagnostic uncertainty information to patients: despite diagnostic uncertainty being extremely common, research into its communication is relatively underdeveloped [61, 62]. There has, however, been increasing interest in recent years: a growing empirical base can now guide decision‐making in this area.

Several studies have identified that doctors are sometimes reluctant to explicitly discuss diagnostic uncertainty due to concerns that it will be negatively perceived or not tolerated by patients, and that it may cause unnecessary (and even harmful) worry [20, 63, 64]. Until recently, a paucity of patient‐focused research made it difficult to determine whether such concerns were justified—there was limited empirical evidence about the effects on patients of communicating diagnostic uncertainty. More recently, a video vignette study that specifically examined the impact of diagnostic uncertainty communication on patients found that vignettes depicting high diagnostic uncertainty communication were more worrying than those depicting low diagnostic uncertainty [65]. The effect size was small—the high diagnostic uncertainty vignettes were perceived as only marginally more worrying—but it lends some credence to concerns that communicating diagnostic uncertainty might worry patients.

There is, however, evidence for benefits to communicating diagnostic uncertainty. In the aforementioned video vignette study, although communicating diagnostic uncertainty was more worrying, the majority of patients still preferred to receive this information [65]. Many patients in this study appreciated greater discussion about uncertainties in investigations and alternate diagnoses being considered, as it facilitated a deeper understanding of their medical situation. In keeping with this, an ethnographic study examining communication in acute settings showed that when doctors shared their thought processes and diagnostic reasoning (including discussion of differential diagnoses), it facilitated greater shared understanding and patient satisfaction [66]. Another study involving patients with endometriosis showed that patients valued the communication of diagnostic uncertainty in facilitating informed decision‐making and fostering trust via perceptions of physician honesty [67].

The idea that patients value being involved and included in the diagnostic process—even when the diagnosis is uncertain, and the diagnoses being considered are potentially worrying—is further supported by research examining patient diagnostic experiences in specific diseases. For example, multiple studies have demonstrated an overall patient preference for open communication about the possibility of multiple sclerosis at the initial point of testing or during work‐up, even when the diagnosis is still only suspected and remains uncertain [68, 69, 70, 71, 72]. Similarly, qualitative research examining patient experiences of motor neurone disease has demonstrated a patient desire to be included and valued in the diagnostic process [73].

Communicating diagnostic uncertainty may also have positive effects upon patient health‐seeking behaviours. Safety‐netting is a well‐established practice by which clinicians share information with patients to help them identify the need to seek further medical help if their condition does not improve [74] it is an important strategy for mitigating against harms associated with diagnostic error. Many have recommended that safety‐netting should include explicit acknowledgement of any diagnostic uncertainty [74, 75, 76, 77]—this may empower patients to appropriately re‐attend by emphasising that the working diagnosis could be incorrect. For example, a study examining primary care diagnostic errors noted that patients did not always understand the provisional nature of the initial working diagnosis [78]; another study examining missed cancer diagnoses highlighted that patients often perceived the initial (ultimately incorrect) benign diagnosis as definitive [37]. Research specifically examining whether communicating diagnostic uncertainty actually makes patients more likely to appropriately reattend—and whether it ultimately improves long‐term health outcomes or reduces harm from diagnostic error—is, however, lacking [79].

Overall, the evidence base remains relatively limited (in particular, there is a need for more patient‐focused research looking specifically at diagnostic uncertainty communication). There are, however, a small number of studies to support the idea that communicating diagnostic uncertainty may have important positive effects for patients.

### 4.2 Effects of *Not* Communicating Diagnostic Uncertainty

In deciding whether to disclose potentially worrying diagnostic uncertainty information, the doctor must also consider the likely consequences of not disclosing this information. In situations when a patient already suspects a ‘worrying’ diagnosis, the decision to not openly discuss it may simply serve to compound existing anxieties; furthermore, patients may simply look up the information themselves at home [65]. Assuming that patients have not already considered certain possible diagnoses can stifle discussion that patients may value—even if the diagnoses being discussed induce worry.

More generally, there is potential for misunderstandings and incorrect assumptions when information is not shared throughout the diagnostic process. That doctors and patients often have divergent ideas about the differential diagnosis has been demonstrated [80], as has the fact that patients commonly have unvoiced concerns about possible diagnoses [81]. In the absence of clear discussion about what diagnoses are being considered, patients may form their own (inaccurate) conclusions, which may even be more worrying that the reality. This is supported by an ethnographic study examining communication around diagnosis in UK acute care settings, which found that when doctors did not explain the reasons for doing investigations, patients sometimes made inaccurate and anxiety‐inducing assumptions about the differential diagnosis [66].

In the context of patients with long‐standing symptoms but no diagnosis (sometimes referred to as medically unexplained symptoms [MUS]), failure to explicitly acknowledge diagnostic uncertainty can also be harmful. Research has demonstrated the challenges of living with the uncertainty of non‐diagnosis, in particular, difficulties with having to justify or defending the legitimacy of their illnesses [82, 83]. Not directly addressing ongoing uncertainty can invalidate patient concerns and foster feelings of rejection or dismissal [84]. For example, in a study examining patients with MUS and their experiences of communication in primary care, the failure of GPs to acknowledge the ongoing uncertainty and the limits of medical understanding was a source of frustration for patients [85].

Finally, we must consider the harms and benefits of communication not just at the level of an individual patient but also at a population level. A full discussion of the extensive literature on the concept of trust in the doctor–patient relationship is beyond the scope of this paper, but it is important to note that effective and open communication has been emphasised as important in building trust [86, 87]. Research examining doctor–patient trust in various contexts has highlighted perceived physician dishonesty as a major barrier to trust [88, 89]. If doctors repeatedly withhold certain sorts of information from their patients, even if it is withheld benignly with the intention of avoiding harmful worry, it could drive a more general loss of trust with the medical profession in the long term.

## 5 Challenges to Diagnostic Uncertainty Communication in Practice

We have argued that even though communicating diagnostic uncertainty can induce worry (which may be considered harmful in some cases), the benefits to the disclosure (and the harms of nondisclosure) often still favour communication of the information.

One problem with using an assessment of consequences to guide communication relates to a general epistemic criticism of consequentialism, termed ‘cluelessness’ by Lenman—we do not know the long‐term consequences of our actions, and therefore, a moral theory that relies on these (unknowable) consequences is unhelpful [90]. In situations of diagnostic uncertainty, at the point at which the doctor has to decide whether to disclosure potentially worrying information, they do not know what the eventual outcomes will be. Two separate elements of uncertainty can be considered: (i) the clinical course (i.e., what the actual diagnosis is) and (ii) how the individual patient will respond to the information being disclosed.

A common response to the cluelessness objection is to shift focus to the reasonable expected consequences of an action, as opposed to the actual consequences [91, 92]. An agent must consider all the possible outcomes of an action, weighing up both the probability of the outcomes occurring and their respective values [93]. As Greaves summarises, ‘[a]n agent subjectively‐ought to perform (roughly) the action that seems most likely to lead to the best consequences, given the agent's beliefs at the time of action’ [94].

This explains why many would support a doctor who gives a patient a medication that has a 99% chance of cure but a 1% chance of severe adverse reaction, even if the patient is very unlucky and experiences the adverse reaction—the expected outcome was one of benefit. In medicine, decision‐making often takes place in conditions of considerable uncertainty and doctors frequently make decisions based upon the expected consequences of their actions. Such judgements are as far as possible evidence‐based—but must acknowledge the fact that the evidence is often imperfect.

Applying this to diagnostic uncertainty communication, the doctor should be guided by their assessment of the reasonable expected outcomes (acknowledging that they cannot know for certain if their predictions will be correct). The accuracy of their predictions is therefore of importance: if doctors' assessments of the expected consequences of communicating diagnostic uncertainty information are inaccurate, this represents a serious problem for this approach. In the previous section, we presented empirical evidence suggesting that the majority of patients will value communication of diagnostic uncertainty—but this preference is not universal, and a minority of patients may not respond so positively to the disclosure of such information [65].

The issue of predicting how individual patients will actually respond to the disclosure of diagnostic uncertainty information is a thorny one, as different patients may not react information in a consistent and predictable manner. The literature exploring whether patient demographic factors can reliably predict informational preferences is conflicting, and various studies have concluded that patient information preferences cannot be predicted from clinical or sociodemographic data alone [95, 96].

An alternative approach is to directly ask patients: by asking them what information they would value, can we better understand—and ultimately predict—the impact of disclosing information to them? Sayers et al. suggested taking an ‘ethical history’, providing hospital inpatients with a questionnaire exploring their preferences regarding truthful disclosure and participation in decision‐making [97]. Other tools have been developed with the intention of better understanding individual patient informational preferences in a range of settings [98, 99, 100, 101, 102]. Such tools are not widely used in clinical practice, and none have been developed to specifically assess patient preferences for diagnostic uncertainty communication. Clinicians often underestimate patients' desire for information and discussion, but overestimate their desire to make decisions [103]. In this context, it is important that tools do not elide preferences for information with the desire to take responsibility for treatment decisions: although patients may value being given information about their medical situation, they may still prefer to take a more passive role in decision‐making.

A more fundamental challenge to the idea of using instruments to guide information disclosure relates to the extent to which patients have informational preferences based on stable values that they can themselves articulate [104]. Epstein has argued for a model of shared deliberation, a collaborative process in which physicians themselves play a role in eliciting exploring, and questioning informational preferences and helping patients construct them [104, 105]. Doctors must effectively engage patients in constructing preferences in the face of complexity, a process that is ‘relational, dynamic, iterative, provisional, and conditional’ [104, 106]. According to such an approach, predicting how patients will respond to the disclosure of diagnostic uncertainty information is likely to require a more involved process than the patient completing a questionnaire at the start of a consultation.

Epstein has also highlighted the need for doctors to guard against the pitfalls of unconscious bias and self‐deception when deciding whether to disclose possibly relevant clinical information [106, 107]. Clinicians may make inaccurate assumptions about what patients want to know based on factors such as perceived education level or may unwittingly have a poor grasp on the patient's values and preferences and instead project their own onto them. There is an inherent paternalism in a doctor assessing the expected consequences of a decision to (not) disclosure potentially worrying information and then weighing up the harms and benefits—the process inevitably involves the doctor making their own assessment about whether the outcomes will be positive or negative for the patient in question. In this context, it is extremely important for doctors to demonstrate self‐awareness, critically assessing and scrutinising their tacit judgements [107].

In summary, a key challenge for communicating within the diagnostic process lies in its inherent uncertainty: at the point of disclosing potentially worrying information, a doctor may not know the actual diagnosis or how the individual patient will respond to the information. This can, to some extent, be mitigated by focusing on the reasonably expected—as opposed to the actual—outcomes. The challenges of accurately predicting how an individual patient will respond to the disclosure of potentially worrying information remain significant. More empirical research may be helpful in improving the accuracy of our predictions, but models that consider the important role that doctors themselves can play in constructing patient information preferences may also prove useful in guiding such communication. The manner in which uncertainty is discussed—taking into account the clinician's style of communication and the context—is likely to be very important in influencing how a patient responds [108]. Further research exploring how doctors might best work with patients to elucidate their information preferences and communicate uncertainty sensitively is required.

## 6 Conclusion

Doctors are often motivated by a desire to avoid causing their patients worry. In this paper, we provide a defence of the disclosure of diagnostic uncertainty information to patients, even if the disclosure induces significant patient worry.

Using a comparative counterfactual account of harm, we argue that worry can be harmful in some—but importantly not all—situations. Worry can sometimes be beneficial: some worry, particularly if it is proportionate to the risk of a future event and is related to a future event over which the patient may have some control, is not harmful.

Even if worry is sometimes harmful, the disclosure of information that induces worry may still be ethically permissible. One way to approach the issue is the application of a broadly consequentialist harm–benefit analysis—considering whether the overall benefits of the disclosure (e.g., improved shared understanding, autonomy and trust in the doctor–patient relationship) outweigh the harms induced by the worry. There is growing empirical evidence to suggest that honest communication within the doctor–patient relationship can be beneficial at both an individual and population level, and that the disclosure of diagnostic uncertainty (including discussion of potentially worrying differential diagnoses) is often valued by patients. We do, however, note some important limitations to this approach: in particular, difficulty in predicting the impact that the disclosure of diagnostic uncertainty information will have on an individual patient.

Ultimately, we argue that minimisation of patient worry should not be the dominant goal of communication within the diagnostic process. Worry is not always harmful, and even if it sometimes is, patients may still want to be told information that worries them. A holistic consideration of the expected consequences of communication—including self‐assessment by the doctor to avoid unwitting bias or unwarranted projection of their own values or views—may often support the communication of diagnostic uncertainty information, *even* if it worries the patient.

## Data Availability

Data sharing is not applicable to this article as no data sets were generated or analysed during the current study.

## Endnotes

1 It is important to acknowledge that the underlying assumption, that the provision of more information results in greater autonomy, has been challenged. Certainly, the provision of information that is overwhelming or overly complex so as to be incomprehensible to a patient will not support their autonomy. We will assume for the rest of this paper that the diagnostic uncertainty information being shared is presented in a manner that is understandable to the patients involved. Although some have raised concerns that patients will be unable (or indeed unwilling) to engage in discussion about diagnostic uncertainty, there is little empirical evidence to support these concerns.
